# Coupled Map Lattice Modeling and Robustness Analysis of Simplicial Complex Networks with Higher-Order Interactions

**DOI:** 10.3390/e28060639

**Published:** 2026-06-05

**Authors:** Luqian Wang, Jun Yin, Xiujuan Ma, Hongyu Chen

**Affiliations:** 1 The College of Computer, Qinghai Normal University, Xining 810016, China; ohh_wlq@163.com (L.W.); qhnumaxiujuan@163.com (X.M.); 202333331030@stu.qhnu.edu.cn (H.C.); 2 The State Key Laboratory of Tibetan Intelligence, Qinghai Normal University, Xining 810008, China

**Keywords:** higher-order networks, simplicial complexes, robustness, cascading failures, CML

## Abstract

Cascading failures in complex networks occur when local node or edge failures propagate to trigger large-scale collapse. Traditional pairwise network models cannot adequately capture group coordination and multi-agent higher-order interactions. Higher-order networks incorporating simplicial structures more accurately represent group and multi-node interactions, providing a new framework to study cascading failures and network robustness. The paper proposes a higher-order coupled map lattice (CML) model to characterize cascading failures in simplicial complex networks and analyze the influence of higher-order structures on network robustness. Further experiments on fourth-order simplicial networks investigate robustness differences under various topologies and attack strategies. Results indicate that fourth-order simplicial networks are vulnerable to targeted attacks but robust against random failures, regardless of network type. Furthermore in single-order networks, the higher simplex dimensions, the greater robustness. The theoretical perturbation thresholds for third-order networks show a negative correlation between the critical perturbation and the sum of network coupling parameters. These results are validated by analysis of simplices added to ordinary networks, destructive experiments, and empirical networks. This study deepens the understanding of cascading failure mechanisms and robustness in higher-order networks, and provides theoretical guidance for designing resilient networks based on higher-order structures.

## 1. Introduction

Cascading failures, as a critical mechanism of failure propagation, are prevalent across power systems, transportation networks, communication networks, and social networks. To date, extensive research has been conducted on cascading failures in complex networks, yielding a substantial body of established results [1,2]. To investigate the propagation and cascading effects of node failures in complex networks, Wang and Xu proposed a cascading failure model based on the coupled map lattice (CML) theory [3,4]. However, traditional network models primarily focus on pairwise connections between nodes [5,6,7,8], which makes them insufficient for capturing the higher-order interactions and high-dimensional relationships that are ubiquitous in real-world systems, including group decision-making, biochemical reactions, neuronal assemblies, and cooperative social behaviors. Compared with traditional networks, higher-order networks incorporate not only one-to-one connections between nodes but also group interactions and high-dimensional relational structures [9]. In recent years, considerable progress has been made in the study of higher-order networks [10,11,12,13,14]. Battiston et al. [9] provide a comprehensive overview of these higher-order network structures, emphasizing the crucial role of higher-order interactions and their impact on network dynamics and functionality. As an important representation of higher-order structures, simplicial-structure-based higher-order networks have been extensively studied in recent years in areas such as synchronization [15,16], spreading processes [17,18], community detection [19], and cascading failures [20], revealing dynamical behaviors distinct from those of traditional networks. In particular, simplicial complexes encode not only group interactions but also their hierarchical inclusion relations, making them suitable for modeling structured multi-level systems. A (k)-simplex represents an irreducible interaction among *k* + 1 nodes that cannot be decomposed into independent pairwise links. This property enables simplicial networks to capture collective behaviors that fundamentally differ from dyadic interactions. Practically, higher-order simplices describe collaborative teams, multi-protein complexes, neuronal cell assemblies, and group contagion mechanisms, thereby offering a more realistic representation of real-world interaction patterns. Moreover, the presence of such higher-level structures in higher-order networks can significantly influence the propagation pathways of cascading failures and the overall resilience of the system. It is of significant theoretical importance and practical relevance to leverage the advantages of simplicial complex networks in characterizing multi-body interactions, to investigate their cascading failure propagation mechanisms, and to analyze the effects of different simplicial structure quantities on network robustness.

In recent years, simplicial complexes have attracted increasing attention as an effective framework for characterizing multi-body interaction dynamics in complex systems. Wang et al. [10] constructed the behavioral layer of group interaction as a higher-order network of simplicial complexes, which more accurately simulates the impact of group interaction on the epidemic threshold and stable state. Meanwhile, the robustness of higher-order networks has also received extensive attention [20,21,22]. Ma et al. [20] conducted simulations of cascading failures in coupled map lattices on scale-free hyper-networks, which was not extended to simplicial structures. Yu et al. [23] considered multivariate coupling relationships among nodes and, by incorporating higher-order structures and realistic load redistribution mechanisms, analyzed the robustness of three classes of networks, including synthetic higher-order networks, conventional networks (graphs), and empirical higher-order networks. Their study is mainly confined to a capacity–load model defined on a single simplicial network, in which the core mechanism focuses on load redistribution triggered by node capacity overload. In the presence of multi-body interactions and higher-order structures, the capacity–load model is insufficient to capture the deeper impact of higher-order interactions on failure propagation. In contrast, the coupled map lattice (CML) model is a representative class of nonlinear dynamical models that characterizes the propagation of perturbations by introducing local maps at network nodes combined with coupling terms. As a result, the CML framework is capable of capturing abrupt collapses induced by higher-order structures under perturbations and revealing the dynamical “collapse mechanisms” of networks, as well as the critical role of higher-order structures in system stability. However, the combination of simplicial complex structures with nonlinear higher-order CML dynamics remains relatively underexplored. Therefore, this study introduces a higher-order CML dynamical model to investigate cascading failures in simplicial complex networks.

In this paper, to investigate the robustness of different higher-order structures under the higher-order CML dynamical model, we construct two types of fourth-order simplicial complex networks based on the interaction relationships among nodes and perform numerical simulations. During the simulation process, the simplicial structures in the two types of networks are systematically adjusted to compare the effects of different simplicial configurations on network robustness under various attack strategies. Higher-order simplicial networks exhibit varying robustness under different attack strategies, and their fault propagation processes differ from those of lower-order structures. This indicates that the dimension of simplices significantly affects network robustness, a pattern that holds across different types of networks. Further analysis shows that, when the same number of higher-order structures is introduced, networks with higher-dimensional simplices display distinct levels of robustness. This structural-level pattern is consistent with the dynamical analysis results of higher-order CML models, providing a theoretical reference for understanding the propagation mechanisms of cascading failures in real complex systems. Moreover, the analysis of perturbation thresholds in third-order simplicial complex networks indicates that the critical perturbation triggering global network failure is associated with the total sum of structural coupling parameters, revealing an intrinsic connection between network topology and dynamical stability.

The remainder of this paper is organized as follows. Section 2 briefly introduces the fundamental theory of higher-order networks and the structural characteristics of simplicial complexes. Based on the coupled map lattice (CML) theory, we construct a CML model for higher-order networks, which is then used to predict the cascading failure processes in such networks. In Section 3, we first employ a higher-order network CML model to numerically simulate cascading failures in fourth-order simplicial complex networks and compare the robustness differences among higher-order structures of varying dimensions. Subsequently, higher-order structures of different dimensions are incorporated into ordinary networks, and the corresponding conclusions are further verified through resilience experiments and numerical simulations. Section 4 derives explicit expressions for the perturbation thresholds that induce global failures in third-order simplicial complex networks, providing a valuable complement for understanding and extending the study of thresholds triggering global collapse in higher-order simplicial networks. Section 5 concludes the paper.

## 2. Methods

The primary representations of higher-order networks are hypergraphs and simplicial networks [9,24,25]. In this study, higher-order interaction data are modeled and analyzed on simplicial networks. Simplicial networks are constructed based on the concept of simplicial complexes from algebraic topology, where simplices are used to represent higher-order interactions among nodes in higher-order networks [24]. In higher-order networks with simplicial complex structures, some nodes participate in multiple simplicial complexes. Consequently, the robustness of the network can be affected when such nodes are subjected to external perturbations.

**Figure 1:**
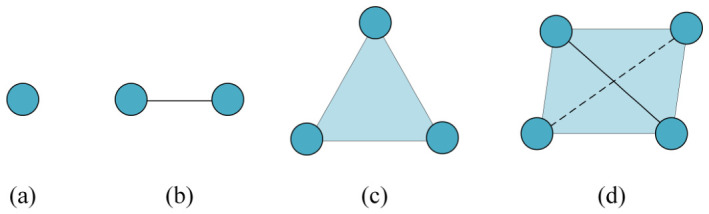
Four types of simplicial structures: (**a**) 0-simplex, (**b**) 1-simplex, (**c**) 2-simplex and (**d**) 3-simplex.

**Figure 2:**
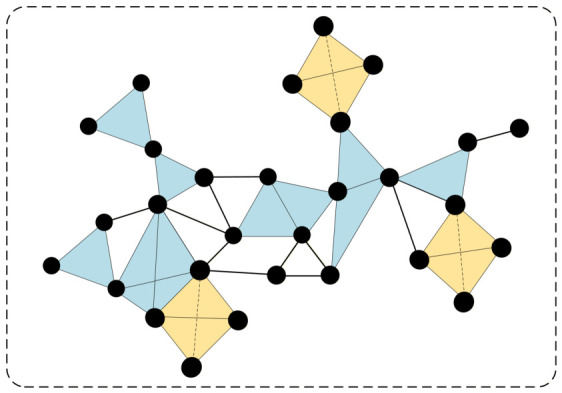
The third-order simplicial complex network contains 29 nodes, 11 1-simplices, 12 2-simplices, and 3 3-simplices. Thick lines represent 1-simplex. The blue surface represents 2-simplex, and the yellow tetrahedron represents 3-simplex. An ordinary triangle only involves pairwise interactions, while a full triangle (2-simplex) additionally includes three-body joint interactions.

Accordingly, a simplicial network can be regarded as a simplicial complex composed of multiple simplices, whose dimension is determined by the order of the highest-dimensional simplex present in the network. A simplicial complex *S*, defined on a node set *V* (|V|=N), is a collection of simplices, $S=\{\alpha_{1},\alpha_{2},\cdots ,\alpha_{m}\}$ such that for any α∈S, every face $\alpha^{\prime }\subseteq \alpha$ is also an element of *S* [26]. For example, when all mutual connections among k+1 nodes are present, a *k*-simplex is formed. A *k*-simplex represents simultaneous interactions among k+1 nodes, and multiple simplices can be connected to form a simplicial complex. Specifically, as illustrated in Figure 1: a 0-simplex is represented by a single node (Figure 1a). A 1-simplex consists of two nodes connected by an edge (Figure 1b), describing pairwise interactions between two nodes. A 2-simplex is represented by a triangle (Figure 1c), capturing simultaneous interactions among three nodes. A 3-simplex is represented by a tetrahedron (Figure 1d), describing simultaneous interactions among four nodes. By extension, higher-order simplices can be constructed in a similar manner. Figure 2 also illustrates an example of a third-order simplicial complex network.

Here, we employ a mathematical representation of simplicial complexes that generalizes the concept of the network adjacency matrix. In simplicial networks, the relationships between nodes and simplicial structures can likewise be represented using matrices. The adjacency matrix *A* of graph G is an N×N matrix, where the matrix element $a_{ij}=1$ if the edge (i,j)∈β exists, and $a_{ij}=0$ otherwise. Accordingly, $A^{\left(1\right)}$ is identical to the conventional adjacency matrix *A* of a standard network. In a simplicial complex network S, for each dimension *m*, an adjacency tensor $A^{\left(m\right)}$ of size $N\times N\times \cdots \times N_{\;m+1}$ can be defined. If the *m*-simplex $\left(i_{1},i_{2},\cdots ,i_{m+1}\right)$ belongs to S, the corresponding tensor element $a_{i_{1},i_{2},\cdots ,i_{m+1}}^{\left(m\right)}=1$, otherwise, it equals 0 [26]. For example, in a 2-simplex, when three nodes i,j and *k* form a complete triangle, $a_{ijk}^{\left(2\right)}=1$, indicating second-order interactions among nodes i,j and *k*; otherwise, $a_{ijk}^{\left(2\right)}=0$. Consequently, in higher-order simplicial networks, each node is influenced by all other nodes within the simplicial structures to which it belongs.

### 2.1. Higher-Order Coupled Map Lattices Model

To explore cascading failures in networks, Wang Xiaofan and Xu Jian [3,4] proposed a successive failure model based on coupled map lattice (CML) theory. In this framework, each node evolves according to a nonlinear local map, while its state is simultaneously influenced by neighboring nodes through coupling terms. The local map characterizes the intrinsic dynamics of an individual unit, whereas the coupling terms represent interactions across the network. Based on this, to investigate more complex cascading effects in networks, we consider a network consisting of *N* nodes with simplicial structures of maximum dimension *m*. We construct a simplicial complex network in which the highest-order simplices are of order *m* (hereafter referred to as a *m*-order simplicial complex network), and based on the coupled map lattice (CML) framework, propose a CML-based cascading failure model for *m*-order simplicial complex networks:

(1) $$\begin{array}{cc} x_{i}\left(t+1\right) & =\left|\left(1-\varepsilon_{1}-\varepsilon_{2}-\cdots -\varepsilon_{m}\right)f\left(x_{i}\left(t\right)\right)+\varepsilon_{1}\sum_{j=1,j\neq i}^{N}f\left(x_{j}\left(t\right)\right)\frac{A_{ij}}{k_{i}^{\left(1\right)}}+\right.\\ \; & \;\left.\frac{\varepsilon_{2}}{2!}\sum_{j,k=1,j,k\neq i}^{N}f\left(x_{j}\left(t\right)\right)f\left(x_{k}\left(t\right)\right)\frac{B_{ijk}}{k_{i}^{\left(2\right)}}+\cdots +\right.\\ \; & \;\left.\frac{\varepsilon_{m}}{m!}\sum_{\begin{array}{c} j_{1},j_{2}\cdots j_{m}=1,\\ j_{1},j_{2}\cdots j_{m}\neq i \end{array}}^{N}f\left(x_{j_{1}}\left(t\right)\right)f\left(x_{j_{2}}\left(t\right)\right)\cdots f\left(x_{j_{m}}\left(t\right)\right)\frac{M_{ij_{1}j_{2}\cdots j_{m}}}{k_{i}^{\left(m\right)}}\right|, \end{array}$$

where $\varepsilon_{1},\varepsilon_{2},\ldots ,\varepsilon_{m}\in \left(0,1\right),\;\left(i=1,2,\ldots ,N\right)$.

Here, $x_{i}\left(t\right)$ denotes the state of node *i* at time *t*, *M* represents the generalized adjacency matrix describing the relationships among nodes in the *m*-order network, $k_{i}^{\left(m\right)}$ denotes the generalized degree of node *i* in the *m*-order network, and $\varepsilon_{1},\varepsilon_{2},\ldots ,\varepsilon_{m}\in \left(0,1\right)$ denote the coupling strengths of the 1st to *m*-order simplices, respectively. Here, each higher-order term is multiplied by $\frac{1}{m!}$ to avoid double-counting the contributions of identical simplices, thereby standardizing each term. The nonlinear function *f* characterizes the intrinsic dynamics of each node. Here, we adopt the chaotic Logistic map function, f(x)=4x(1−x). When 0≤x≤1, it follows that 0≤f(x)≤1. The absolute value in Equation (1) ensures that the state of each node remains non-negative.

We define that in a *m*-th order simplicial complex network, if there exists at least one *m*-simplex in which m+1 nodes interact, the corresponding element in the generalized adjacency matrix is 1, otherwise, it is 0. The dimensionality of generalized adjacency matrices for higher-order structures is usually very high, making direct analysis computationally, storage-wise, and modeling-wise challenging. Here, we employ the approach mentioned in Section 2.1, representing the adjacency of a *m*-th order simplicial complex as the tensor $A^{\left(m\right)}$:

(2) $$A_{ij}^{\left(m\right)}=\frac{1}{(m-1)!}\sum_{\begin{array}{c} j_{1},j_{2}\ldots j_{m}=1,\\ j_{1},j_{2}\ldots j_{m}\neq i \end{array}}^{N}M_{ij_{1}j_{2}\ldots j_{m}}.$$

For example, let $A_{ij}^{\left(2\right)}$ be the second-order adjacency matrix, then $A_{ij}^{\left(2\right)}=\sum_{k=1}^{N}B_{ijk}$, where $B_{ijk}$ is the second-order generalized adjacency matrix with respect to node *k* that represents the second-order interaction among nodes *i*, *j* and *k*. In a 2-simplex, the three nodes *i*, *j* and *k* act within the same second-order simplex, where node *i* is influenced by both nodes *j* and *k*. $B_{ijk}$ is defined as 1 if and only if nodes *i*, *j* and *k* participate in a second-order interaction; otherwise, $B_{ijk}=0$. For $B_{ijk}=0$, the nodes may only have first-order connections or no direct interactions at all (e.g., isolated nodes). Additionally, during the computation in Equation (1), $B_{ijk}$ is appropriately normalized to avoid double-counting the same 2-simplex.

In simplicial complex networks, we define the generalized degree of a node as the number of distinct simplices of any dimension to which the node is connected. In such networks, the generalized degree of a node is also related to the higher-order adjacency matrix $M_{ij_{1}j_{2}\ldots j_{m}}$ in which it participates [27]. We next discuss the generalized degree $k_{i}^{\left(m\right)}$ in higher-order networks:

(3) $$k_{i}^{\left(m\right)}=\frac{1}{m!}\sum_{j_{1}j_{2}\ldots j_{m}=1}^{N}M_{ij_{1}j_{2}\ldots j_{m}},$$

we obtain:

(4) $$k_{i}^{\left(m\right)}=\frac{1}{m!}\sum_{j_{1}=1}^{N}\sum_{j_{2}=1}^{N}\cdots \sum_{j_{m}=1}^{N}M_{i,j_{1},j_{2},\cdots ,j_{m}}^{\left(m\right)}.$$

Assuming all interactions are unweighted and undirected, for 2-simplices, the generalized degrees of node *i* are given by $k_{i}^{\left(1\right)}=\sum_{j=1}^{N}M_{ij}^{\left(1\right)}$ and $k_{i}^{\left(2\right)}=\frac{1}{2}\sum_{j,k=1}^{N}B_{ijk}=\frac{1}{2}\sum_{j=1}^{N}\sum_{k=1}^{N}M_{ijk}^{\left(2\right)}$. The degree $k_{i}$ of node *i* represents the total number of simplices associated with it, i.e., $k_{i}=k_{i}^{\left(0\right)}+k_{i}^{\left(1\right)}+k_{i}^{\left(2\right)}$, for a connected network, $k_{i}^{\left(0\right)}=0$.

Additionally, we can define the generalized *m*-order degree $k_{ij}^{\left(m\right)}$ as the number of *m*-dimensional simplices containing the edge (i,j), which can also be represented using the *m*-dimensional adjacency tensor $A^{\left(m\right)}$:

(5) $$k_{ij}^{\left(m\right)}=\frac{1}{(m-1)!}\sum_{j_{1}=1}^{N}\sum_{j_{2}=1}^{N}\cdots \sum_{j_{m-1}=1}^{N}A_{i,j,j_{1},j_{2},\cdots ,j_{m-1}}^{\left(m\right)}.$$

Thus, $k_{ij}^{\left(1\right)}=A_{ij}^{\left(1\right)}$, while $k_{ij}^{\left(2\right)}$ counts the number of 2-simplices that edge (i,j) participates in, i.e., $k_{ij}^{\left(2\right)}=A_{ijk}^{\left(2\right)}$, and so on.

### 2.2. Cascading Failure Evolution

Since $x_{i}\left(t\right)$ denotes the state of node *i* at time *t*, the state of this node depends not only on the other *m* nodes that belong to the same *m*-order simplicial structure, but also on the states of these *m* nodes at time t−1. If the state of a node at time *t* is known, Equation (1) can be used to evolve the state of node *i* at time t+1. If the state of node *i* remains within the interval (0,1), the node is considered to be in a normal state; if $x_{i}\left(t\right)\geq 1$, node *i* is considered to have failed at time *t*. Moreover, to ensure that all states remain non-negative, absolute values are applied to both sides of Equation (1). It can be seen that when the network contains only simplicial structures of order m≤1, model (1) reduces to the standard CML model for ordinary networks [3,4]:

(6) $$x_{i}\left(t\right)=\left|\left(1-\varepsilon_{1}\right)f\left(x_{i}\left(t-1\right)\right)+\varepsilon_{1}\sum_{\begin{array}{c} j=1\\ j\neq i \end{array}}^{N}f\left(x_{j}\left(t-1\right)\right)\frac{A_{ij}}{k_{i}^{\left(1\right)}}\right|,\;\varepsilon_{1}\in \left(0,1\right).$$

To investigate the cascade failure process in a *m*-order simplicial complex network after a node is attacked, we assume that all nodes in the initial network are in a normal state, i.e., $0<x_{i}\left(t\right)<1$ for i=1,2,⋯,N and t=1,2,⋯,s−1. Suppose node *c* fails at time step *s*, where an external perturbation *R*(R≥1) is applied to node *c*, causing its state $x_{i}\left(s\right)\geq 1$. The state of node *c* can be expressed as:

(7) $$\begin{array}{cc} x_{c}\left(s\right)= & |\left(1-\varepsilon_{1}-\varepsilon_{2}-\cdots -\varepsilon_{m}\right)f\left(x_{i}\left(s-1\right)\right)+\varepsilon_{1}\sum_{\begin{array}{c} j=1\\ j\neq i \end{array}}^{N}f\left(x_{j}\left(s-1\right)\right)\frac{A_{ij}}{k_{i}^{\left(1\right)}}+\\ \; & \frac{\varepsilon_{2}}{2!}\sum_{\begin{array}{c} j,k=1\\ j,k\neq i \end{array}}^{N}f\left(x_{j}\left(s-1\right)\right)f\left(x_{k}\left(s-1\right)\right)\frac{B_{ijk}}{k_{i}^{\left(2\right)}}+\cdots +\\ \; & \frac{\varepsilon_{m}}{m!}\sum_{\begin{array}{c} j_{1},j_{2}\cdots j_{m}=1\\ j_{1},j_{2}\cdots j_{m}\neq i \end{array}}^{N}f\left(x_{j_{1}}\left(s-1\right)\right)f\left(x_{j_{2}}\left(s-1\right)\right)\cdots f\left(x_{j_{2}}\left(s-1\right)\right)\frac{M_{ij_{1}j_{2}\cdots j_{m}}}{k_{i}^{\left(m\right)}}|+R, \end{array}$$

where $\varepsilon_{1},\varepsilon_{2},\ldots ,\varepsilon_{m}\in \left(0,1\right),\;\left(i=1,2,\ldots ,N\right),\;R\geq 1$.

**Figure 3:**
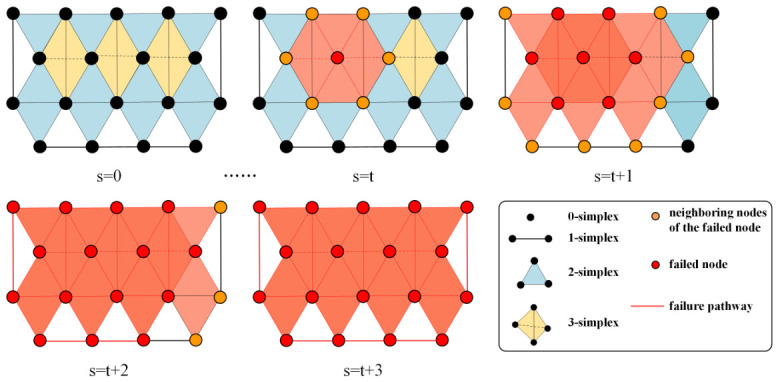
Cascading failure dynamics in a 3-order simplicial complex. Panels from left to right depict the network states at successive time steps s=0,…,s=t,s=t+1,s=t+2, and s=t+3. Red coloring indicates failed nodes and the failure propagation pathway, while orange dots represent the neighbors of the initially failed vertex. The dark red surfaces indicate the failed areas, while the light red surfaces represent the dangerous areas associated with the failed nodes. This schematic illustrates how a single node failure spreads through higher-order structures, leading to a global cascading failure.

Here, in Equation (3), an external perturbation *R* is applied to the state of node *i* to induce failure. That is, $x_{i}\left(t\right)>1$ indicates that node *i* fails at time *s*. In subsequent time steps t+1,t+2,…, the state of node *i* is set to $x_{i}\left(s\right)\equiv 0$. At time t+1, other nodes within the same simplicial structure as node *i* experience state changes due to the influence from node *i*. If a node’s state remains below 1, it is considered normal at time t+1. If it exceeds 1, the node is considered failed at time t+1. Consequently, as time progresses and nodes influence one another, the state of each node evolves, leading to the continuous propagation of cascade failures until global failure occurs in the higher-order network. The external perturbation $R_{c}$ that triggers global failure is defined as the perturbation threshold. Figure 3 simulates the cascading failure process of a small third-order simplex complex network.

For Equation (5), when m=4, the cascading failure model for a higher-order network with a maximum simplex order of 4 can be obtained as follow:

(8) $$\begin{array}{cc} x_{i}\left(t\right)= & |\left(1-\varepsilon_{1}-\varepsilon_{2}-\varepsilon_{3}-\varepsilon_{4}\right)f\left(x_{i}\left(t-1\right)\right)+\varepsilon_{1}\sum_{\begin{array}{c} j=1\\ j\neq i \end{array}}^{N}f\left(x_{j}\left(t-1\right)\right)\frac{A_{ij}}{k_{i}^{\left(1\right)}}+\\ \; & \frac{\varepsilon_{2}}{2!}\sum_{\begin{array}{c} j,k=1\\ j,k\neq i \end{array}}^{N}f\left(x_{j}\left(t-1\right)\right)f\left(x_{k}\left(t-1\right)\right)\frac{B_{ijk}}{k_{i}^{\left(2\right)}}+\\ \; & \frac{\varepsilon_{3}}{3!}\sum_{\begin{array}{c} j,k,l=1\\ j,k,l\neq i \end{array}}^{N}f\left(x_{j}\left(t-1\right)\right)f\left(x_{k}\left(t-1\right)\right)f\left(x_{l}\left(t-1\right)\right)\frac{C_{ijkl}}{k_{i}^{\left(3\right)}}+\\ \; & \frac{\varepsilon_{4}}{4!}\sum_{\begin{array}{c} j,k,l,m=1\\ j,k,l,m\neq i \end{array}}^{N}f\left(x_{j}\left(t-1\right)\right)f\left(x_{k}\left(t-1\right)\right)f\left(x_{l}\left(t-1\right)\right)f\left(x_{m}\left(t-1\right)\right)\frac{D_{ijklm}}{k_{i}^{\left(4\right)}}|+R, \end{array}$$

where $\varepsilon_{1},\varepsilon_{2},\varepsilon_{3},\varepsilon_{4}\in \left(0,1\right),\;R\geq 1$.

To validate Equation (4), we simulate the cascade failure process in a network with a maximum simplex order of 4, and use Equation (8) to model the state of node *i* at time *t*.

## 3. Robustness Analysis of Simplicial Complex Networks and Simulation Experiments

To investigate the cascading process in higher-order networks under different attack strategies, as well as the robustness of simplicial structures of varying dimensions, we applied the fourth-order simplicial complex network CML model (Equation (6)) to two types of fourth-order simplicial complex networks constructed from scale-free and random networks. Cascading failure simulations were then performed on both network types under random and targeted attacks. In the scale-free network-based fourth-order simplicial complex network, the number of simplicial structures is set to $M=N-k_{0}+1$, and all simplex types are generated with equal probability, i.e., $p_{1}=p_{2}=p_{3}=p_{4}$. For the random network-based fourth-order simplicial complex network, M=500, with the initial proportions of the four types of simplices set to 1:1:1:1. In the random attack scenario, a node is selected at random for removal, whereas in the targeted attack scenario, the node with the highest generalized degree is attacked. To ensure experimental stability, the results were averaged over more than one hundred simulation runs. Relevant experimental parameters, their meanings, and corresponding values are listed in Table 1, Table 2 and Table 3.

### 3.1. Robustness Analysis in the Fourth-Order Simplicial Complex Networks

#### 3.1.1. Robustness Analysis of Higher-Order Structure with Attack Strategies

**Figure 4:**
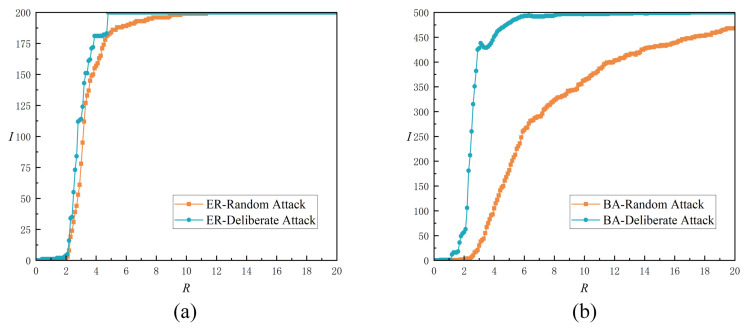
(**a**) Experimental data for fourth-order simplicial complex networks constructed from random networks; (**b**) experimental data for fourth-order simplicial complex networks constructed from scale-free networks.

Both fourth-order simplicial complex networks generated from scale-free networks and those generated from random networks, like conventional networks, exhibit both robust and fragile properties when subjected to external attacks. The results shown in the figure indicate that under both random and targeted attack strategies, the network eventually experiences global failure once the perturbation threshold reaches a certain level. It is clearly observed that, in fourth-order simplicial complex networks, the critical perturbation threshold $R_{c}$ required to trigger global system failure is lower under targeted attacks than under random attacks. Moreover, the propagation of cascading failures under random attacks is slower compared to targeted attacks. This indicates that higher-order networks are more robust against random attacks but more vulnerable to targeted attacks, meaning their ability to withstand random perturbations exceeds that against directed attacks on high-impact nodes. This phenomenon can be attributed to the cascading failure propagation mechanism in higher-order networks: when a node fails, the failure first spreads among the nodes that jointly participate in the same higher-order simplex. Once the entire structure fails, the failure may even propagate to higher-dimensional structures that include the node. Therefore, during targeted attacks, the preferentially selected nodes typically have higher generalized higher-order degrees and participate in many higher-order simplices. The failure of such nodes simultaneously affects multiple higher-order structures, leading to a rapid increase in the number of failed nodes in the network. In contrast, under random attacks, since the node degree distribution in scale-free networks follows a power-law, the randomly selected nodes are mostly low-degree nodes that participate in relatively few higher-order structures. Consequently, the number of failed nodes increases more gradually with perturbation intensity. As a result, the relationship between failed node count and perturbation intensity in scale-free higher-order networks differs significantly from that observed in random higher-order networks under the same attack strategies. Figure 4 below illustrates the relationship between the number of failed nodes *I* and the perturbation threshold *R* for fourth-order simplicial complex networks of the same size under two attack strategies.

#### 3.1.2. Robustness Analysis of Higher-Order Structures with Different Dimensions

In first-order simplicial networks, namely conventional complex networks, nodes are connected solely through edges, and higher-order interactions are relatively weak. Therefore, this section focuses on structures with more pronounced higher-order interactions, specifically higher-order simplicial networks with dimension m≥2. To investigate the robustness of higher-order structures with different dimensions, we construct both *m*-order random simplicial complex networks and *m*-order scale-free simplicial complex networks (with m∈{2,3,4}), and subject nodes in both network types to random and targeted attacks. By comparing the failure evolution processes and the corresponding critical perturbation thresholds under different attack strategies, we analyze and contrast the robustness of higher-order structures across different dimensions. Subsequently, simplicial structures of different dimensions and quantities are incorporated into conventional networks to conduct comparative experiments, thereby examining the effects of both the dimension and the number of higher-order structures on network robustness. In addition, robustness experiments independent of the dynamical evolution of the higher-order CML model are performed to eliminate potential influences of model dynamics on the experimental results. Finally, cascading failure experiments are conducted on empirical network data within the framework of the higher-order CML model, thereby validating the theoretical analysis and simulation results obtained above.

**Figure 5:**
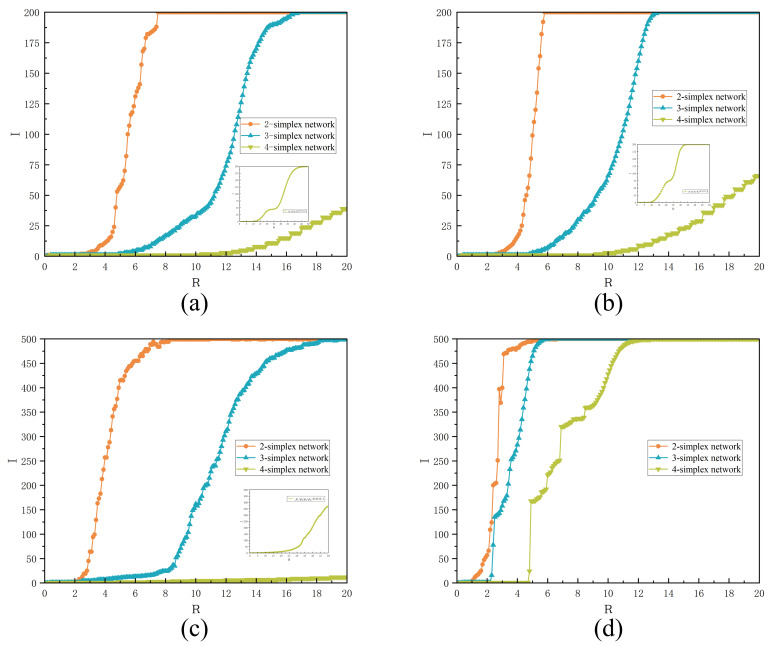
(**a**,**b**) correspond to three types of networks constructed from random networks, whereas panels (**c**,**d**) correspond to three types of networks constructed from scale-free networks. (**a**,**c**) show the results under random attacks on network nodes, while (**b**,**d**) present the results under targeted attacks. The insets illustrate the variation in the number of failed nodes in fourth-order simplicial networks as the external perturbation *R* increases up to 50.

We based on random networks and scale-free networks, we construct six types of networks that contain only a single dimension of simplicial structures: second-, third-, and fourth-order random simplicial networks, as well as their corresponding scale-free simplicial networks. Numerical simulations are then conducted on these six networks. As shown clearly in the figure, regardless of network type, under both random and targeted attack scenarios, node failures occur in second-order simplicial networks even at relatively low perturbation thresholds. As the perturbation intensity further increases, the network eventually approaches global failure. However, compared with networks containing higher-dimensional simplicial structures, second-order simplicial networks exhibit the smallest global failure threshold and the steepest failure curves, indicating greater vulnerability under both attack strategies. From the overall cascading failure curves, it can be observed that second-order simplicial networks display the fastest failure propagation, suggesting a weaker ability to resist external perturbations compared with higher-order structures. Therefore, when a network contains only a single type of simplicial structure (as shown in Figure 5), higher-order simplices require stronger perturbations to trigger global failure, exhibit slower failure propagation, and correspond to larger critical perturbation thresholds for global collapse. Under the same external perturbation, networks with higher-order structures may experience only partial node failures rather than immediate global collapse. Only when the perturbation intensity exceeds a certain critical value do the nodes in the network collectively trigger global failure. These observations indicate that stronger higher-order interactions within higher-dimensional simplicial structures enhance network redundancy and robustness.

**Figure 6:**
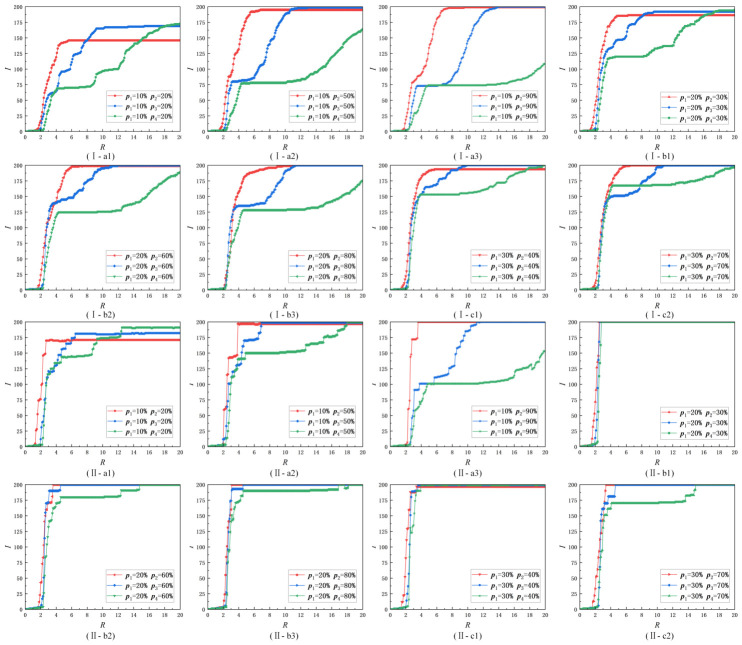
Based on random higher-order networks. Subfigure (**I**) illustrates random attacks on network nodes, while Subfigure (**II**) illustrates targeted attacks on network nodes.

**Figure 7:**
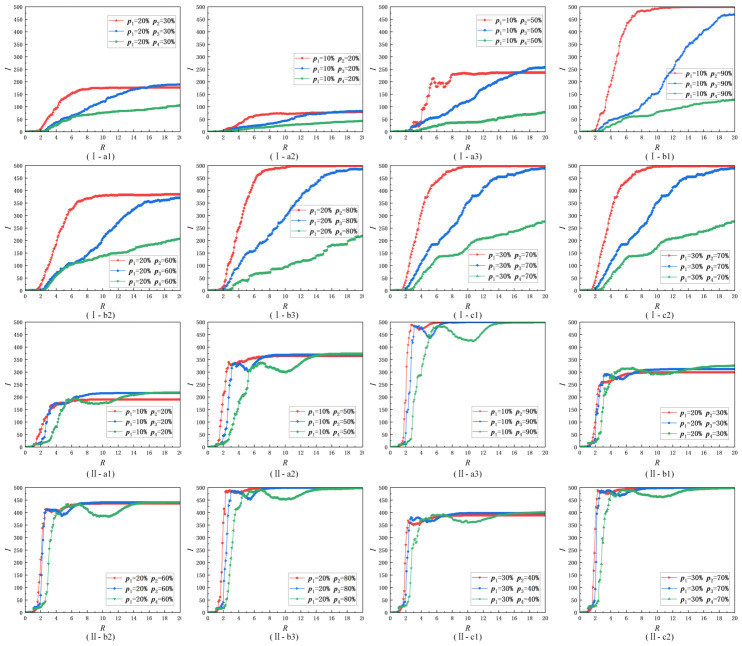
Based on scale-free higher-order networks. Subfigure (**I**) illustrates random attacks on network nodes, while Subfigure (**II**) illustrates targeted attacks on network nodes.

To further validate the robustness of higher-order structures with different dimensions, firstly we introduces simplicial structures of different dimensions into random networks and scale-free networks that originally contain only edge-based connections, and conducts comparative validation experiments. To reduce the influence of lower-order structural robustness on the results, only scenarios dominated by higher-order structures are considered, by imposing that the generation probabilities of higher-order simplices satisfy $p_{2},p_{3},p_{4}>p_{1}$ with $p_{1}<0.3$. The experimental results show that, under the condition of introducing the same number of simplicial structures, regardless of network type and whether random or targeted attack strategies are employed, higher-dimensional simplices lead to slower failure propagation as the external perturbation intensity increases, indicating stronger robustness of higher-dimensional simplicial structures. (In Figure 6 and Figure 7: Dependence of the number of failed nodes *I* on the external perturbation *R* for networks with different proportions of higher-order structures added to random and scale-free networks. Red, blue, and green curves represent networks with different proportions of 2-simplices, 3-simplices, and 4-simplices, respectively.)

**Figure 8:**
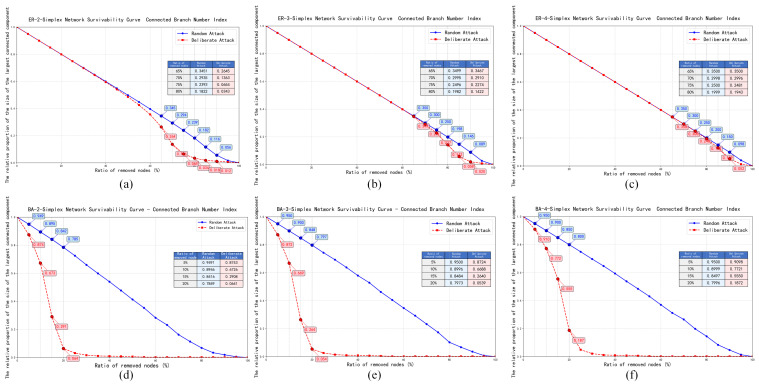
The relationship between the number of removed nodes and the relative size of the network’s largest connected component. The first row of panels (**a**–**c**) shows robustness experiments based on random higher-order networks, while the second row of panels (**d**–**f**) shows experiments based on scale-free networks. The blue curves represent the results under random node attacks, and the red curves represent those under targeted attacks. In the results for random higher-order networks, the tables indicate the relative sizes of the largest connected component when the proportion of removed nodes is 65%, 70%, 75%, and 80% under random attacks (light blue boxes) and targeted attacks (red boxes). In the results for scale-free higher-order networks, the tables show the relative sizes of the largest connected component when the proportion of removed nodes is 5%, 10%, 15%, and 20% under random attacks (light blue boxes) and targeted attacks (red boxes).

Furthermore, in the robustness experiments, to eliminate the influence of dynamics, we did not consider the evolution process of the higher-order CML model. Instead, we gradually removed a certain number of nodes and examined the relative size of the largest connected component with respect to the original network. Accordingly, as the number of removed nodes increases, the relative size of the network’s largest connected component exhibits a monotonically non-increasing trend (in Figure 8). The results indicate that, for networks with any type of simplicial structure, under the same number of node removals, the size of the largest connected component under random attacks was consistently larger than under targeted attacks. This demonstrates that higher-order networks possess stronger structural robustness against random attacks. Furthermore, a comparison among simplicial networks of different dimensions shows that, for the same proportion of node removals, the relative size of the largest connected component in higher-order simplicial networks is consistently larger than in lower-order networks. This indicates that higher-order structures have an advantage in maintaining overall network connectivity. Therefore, in *m*-order (m≥2) simplicial networks, higher-order structures exhibit stronger robustness. This is consistent with the dynamical attack simulation results based on the higher-order CML model in the previous section, further confirming that higher-order simplices provide superior network robustness.

### 3.2. Experiments on Real-World Networks

**Figure 9:**
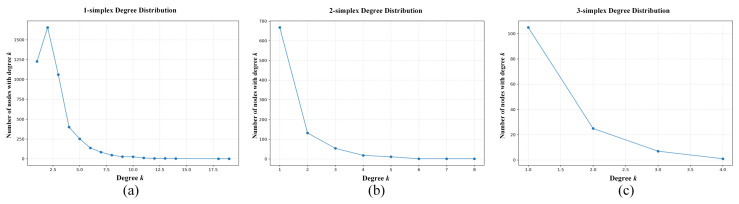
The generalized degree distributions of different-order structures in the H-PUG network.

**Figure 10:**
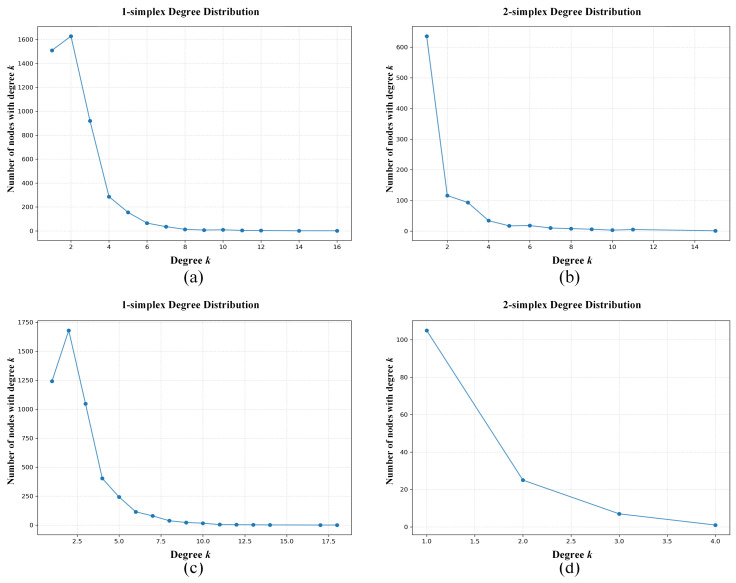
The generalized degree distributions of different-order structures in the 12H-PUG and 13H-PUG networks. (**a**,**b**) show the first-order (edge) and second-order (triangle) degree distributions of the 12H-PUG network, while (**c**,**d**) show the first-order (edge) and third-order (tetrahedron) degree distributions of the 13H-PUG network.

To further validate the conclusions and practical feasibility of the higher-order CML model, we selected an empirical dataset of the Power-US-Grid network from https://networkrepository.com/ (accessed on 25 September 2025). Based on the original standard PUG network, nodes and edges with interactions were fitted into higher-order structures. During this fitting process, it was found that the network could be constructed up to a third-order simplicial complex. Consequently, we built the H-PUG network (retaining all simplicial structures in the network), the 12H-PUG network (containing only first- and second-order simplicial structures), and the 13H-PUG network (containing only first- and third-order simplicial structures). Table 4 reports the numbers of simplices of different dimensions in the three networks. Figure 9 and Figure 10 illustrate the generalized degree distributions of nodes associated with different higher-order structures in the three networks. The results indicate that the generalized degree distributions of nodes in all three networks follow a power-law behavior. Therefore, the H-PUG network can be classified as a scale-free higher-order network. Based on this, CML cascade failure experiments were conducted on the H-PUG, 12H-PUG, and 13H-PUG networks to comparatively analyze the robustness of different higher-order networks.

**Figure 11:**
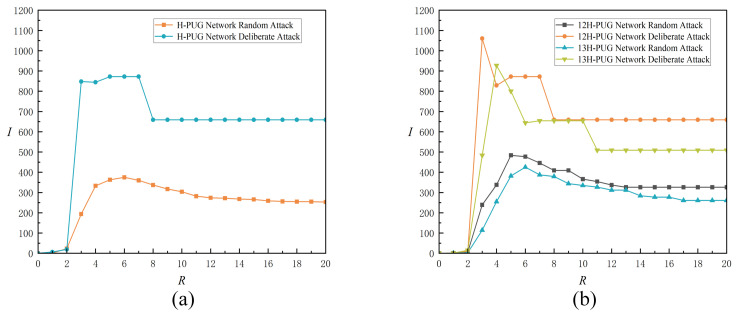
(**a**) Number of failed nodes (*I*) versus external perturbation strength (*R*) in the H-PUG network under different attack strategies. (**b**) Number of failed nodes (*I*) as a function of *R* for the 12H-PUG and 13H-PUG networks under different attack strategies.

Figure 11 below illustrates the variation of the number of failed nodes *I* with the perturbation magnitude *R* under different attack strategies for the three types of PUG networks. The results indicate that, under both attack strategies, the overall trend of *I* with increasing *R* is monotonically non-decreasing: within the small perturbation range, *I* increases rapidly with *R*; once the perturbation reaches a certain critical range, the failure size peaks, and as the perturbation further increases, the number of failed nodes gradually decreases and stabilizes.

In Figure 11a, the cascading failure processes of the H-PUG network under different attack strategies are compared. The results indicate that, compared with random attacks, targeted attacks can induce large-scale node failures under much smaller perturbations, implying a significantly lower level of network robustness. This observation further validates the conclusion drawn in Section 3.1.2 that scale-free higher-order networks are more vulnerable to targeted attacks than to random attacks under different attack strategies. Moreover, throughout the perturbation range, the failure evolution curves under random and targeted attacks are clearly separated, further indicating that the attack strategy significantly affects the network’s failure mode and the intensity of cascade propagation.

By comparing the 12H-PUG and 13H-PUG networks (Figure 11b), it can be observed that under the same perturbation strength, the scale of failed nodes differs significantly across network structures. This demonstrates that network topology has a crucial influence on the cascade failure process and further validates the conclusion that higher-order structures possess stronger robustness. Owing to the intrinsic properties of real-world networks and their large scale, the external perturbations considered here may not necessarily trigger global network failure. Nevertheless, they can still exert substantial impacts on the overall network operation.

In this section, we theoretically analyze the perturbation threshold for cascading failures in third-order simplicial complex networks based on the average generalized degree, and derive an expression for the threshold. According to the cascading failure model of third-order simplicial complex networks given in Equation (8), if a node *c* in the hypernetwork is attacked at time *t*, then at time t+1, in order for all nodes in the third-order simplicial complex network to fail, the following condition must be satisfied.

## 4. Analysis of the Perturbation Threshold **R**

(9) $$\begin{array}{cc} x_{i}\left(t+1\right)= & \left|\left(1-\varepsilon_{1}-\varepsilon_{2}-\varepsilon_{3}\right)f\left(x_{i}\left(t\right)\right)+\varepsilon_{1}\sum_{\begin{array}{c} j=1,j\neq i \end{array}}^{N}f\left(x_{j}\left(t\right)\right)\frac{A_{ij}}{k_{i}^{\left(1\right)}}\right.\\ \; & +\frac{\varepsilon_{2}}{2!}\sum_{\begin{array}{c} j,k=1,j,k\neq i \end{array}}^{N}f\left(x_{j}\left(t\right)\right)f\left(x_{k}\left(t\right)\right)\frac{B_{ijk}}{k_{i}^{\left(2\right)}}\\ \; & +\left.\frac{\varepsilon_{3}}{3!}\sum_{\begin{array}{c} j,k,l=1,j,k,l\neq i \end{array}}^{N}f\left(x_{j}\left(t\right)\right)f\left(x_{k}\left(t\right)\right)f\left(x_{l}\left(t\right)\right)\frac{C_{ijkl}}{k_{i}^{\left(3\right)}}\right|\geq 1, \end{array}$$

$\varepsilon_{1},\varepsilon_{2},\varepsilon_{3}\in \left(0,1\right)$, i=1,2,…,N,i≠c.

In the above equation, $A_{ij}$, $B_{ijk}$ and $C_{ijkl}$ denote the adjacency relationships among any two, three, or four nodes in a third-order simplicial complex network. Specifically, if nodes *i* and *j* (or i,j,k, or i,j,k,l) belong to the same simplex, then $A_{ij}=1$ ($B_{ijk}=1$, $C_{ijkl}=1$). Otherwise, $A_{ij}=0$ ($B_{ijk}=0$, $C_{ijkl}=0$). Therefore, when computing the state of node *i*, only the terms with $A_{ij}=1$ ($B_{ijk}=1$, $C_{ijkl}=1$) are retained in Equation (9).

When node *c* is attacked at time step *t*, $x_{c}\left(t\right)\geq 1$, and thus:

(10) $$f\left(x_{c}\left(t\right)\right)=4x_{c}\left(t\right)\left(1-x_{c}\left(t\right)\right)=-4x_{c}\left(t\right)\left(x_{c}\left(t\right)-1\right)\leq 0.$$

Let ${\bar{x}}_{c}\left(t\right)$ denote the state of node *c* at time step *t* in the absence of external perturbation *R*, then $0<{\bar{x}}_{c}\left(t\right)<1$. By applying the properties of absolute inequalities and Equation (10), we obtain the following form:

(11) $$\begin{array}{cc} \; & \left(1-\varepsilon_{1}-\varepsilon_{2}-\varepsilon_{3}\right)f\left(x_{c}\left(t\right)\right)+\varepsilon_{1}\sum_{\begin{array}{c} j=1,j\neq c \end{array}}^{N}f\left(x_{j}\left(t\right)\right)\frac{A_{cj}}{k_{c}^{\left(1\right)}}\\ \; & +\frac{\varepsilon_{2}}{2!}\sum_{\begin{array}{c} j,k=1,j,k\neq c \end{array}}^{N}f\left(x_{j}\left(t\right)\right)f\left(x_{k}\left(t\right)\right)\frac{B_{cjk}}{k_{c}^{\left(2\right)}}\\ \; & +\frac{\varepsilon_{3}}{3!}\sum_{\begin{array}{c} j,k,l=1,j,k,l\neq c \end{array}}^{N}f\left(x_{j}\left(t\right)\right)f\left(x_{k}\left(t\right)\right)f\left(x_{l}\left(t\right)\right)\frac{C_{cjkl}}{k_{c}^{\left(3\right)}}\geq 1, \end{array}$$

or

(12) $$\begin{array}{cc} \; & \left(1-\varepsilon_{1}-\varepsilon_{2}-\varepsilon_{3}\right)f\left(x_{i}\left(t\right)\right)+\varepsilon_{1}\sum_{\begin{array}{c} j=1,j\neq i \end{array}}^{N}f\left(x_{j}\left(t\right)\right)\frac{A_{ij}}{k_{i}^{\left(1\right)}}\\ \; & +\frac{\varepsilon_{2}}{2!}\sum_{\begin{array}{c} j,k=1,j,k\neq i \end{array}}^{N}f\left(x_{j}\left(t\right)\right)f\left(x_{k}\left(t\right)\right)\frac{B_{ijk}}{k_{i}^{\left(2\right)}}\\ \; & +\frac{\varepsilon_{3}}{3!}\sum_{\begin{array}{c} j,k,l=1,j,k,l\neq i \end{array}}^{N}f\left(x_{j}\left(t\right)\right)f\left(x_{k}\left(t\right)\right)f\left(x_{l}\left(t\right)\right)\frac{C_{ijkl}}{k_{i}^{\left(3\right)}}\leq -1. \end{array}$$

where $\varepsilon_{1},\varepsilon_{2},\varepsilon_{3}\in \left(0,1\right)$, the left-hand side of Equation (11) can be transformed as follows:

$$\begin{array}{c} f\left(x_{i}\left(t\right)\right)+\frac{\varepsilon_{1}}{k_{i}^{\left(1\right)}}\left[\sum_{\begin{array}{c} j=1,j\neq i \end{array}}^{N}\left(f\left(x_{j}\left(t\right)\right)-k_{i}^{\left(1\right)}f\left(x_{i}\left(t\right)\right)\right)\right]\\ \;+\frac{\varepsilon_{2}}{k_{i}^{\left(2\right)}}\left[\frac{1}{2!}\sum_{\begin{array}{c} j,k=1,j,k\neq i \end{array}}^{N}\left(f\left(x_{j}\left(t\right)\right)f\left(x_{k}\left(t\right)\right)-k_{i}^{\left(2\right)}f\left(x_{i}\left(t\right)\right)\right)\right]\\ \;+\frac{\varepsilon_{3}}{k_{i}^{\left(3\right)}}\left[\frac{1}{3!}\sum_{\begin{array}{c} j,k,l=1,j,k,l\neq i \end{array}}^{N}\left(f\left(x_{j}\left(t\right)\right)f\left(x_{k}\left(t\right)\right)f\left(x_{l}\left(t\right)\right)-k_{i}^{\left(3\right)}f\left(x_{i}\left(t\right)\right)\right)\right]\\ =f\left(x_{i}\left(t\right)\right)+\frac{\varepsilon_{1}}{k_{i}^{\left(1\right)}}\left\{\sum_{\begin{array}{c} j=1,j\neq i \end{array}}^{N}\left(f\left(x_{j}\left(t\right)\right)-k_{i}^{\left(1\right)}f\left(x_{i}\left(t\right)\right)+\left[f\left({\bar{x}}_{c}\left(t\right)\right)-f\left(x_{c}\left(t\right)\right)\right]\right.\right.\\ \;\left.\left.+\left[f\left(x_{c}\left(t\right)\right)-f\left({\bar{x}}_{c}\left(t\right)\right)\right]\right)\right\}\\ \;+\frac{\varepsilon_{2}}{k_{i}^{\left(2\right)}}\left\{\frac{1}{2!}\sum_{\begin{array}{c} j,k=1,j,k\neq i \end{array}}^{N}\left(f\left(x_{j}\left(t\right)\right)f\left(x_{k}\left(t\right)\right)-k_{i}^{\left(2\right)}f\left(x_{i}\left(t\right)\right)\right)\right.\\ \;\left.+\left[f\left({\bar{x}}_{c}\left(t\right)\right)-f\left(x_{c}\left(t\right)\right)\right]+\left[f\left(x_{c}\left(t\right)\right)-f\left({\bar{x}}_{c}\left(t\right)\right)\right]\right\}\\ \;+\frac{\varepsilon_{3}}{k_{i}^{\left(3\right)}}\left\{\frac{1}{3!}\sum_{\begin{array}{c} j,k,l=1,j,k,l\neq i \end{array}}^{N}\left(f\left(x_{j}\left(t\right)\right)f\left(x_{k}\left(t\right)\right)f\left(x_{l}\left(t\right)\right)-k_{i}^{\left(3\right)}f\left(x_{i}\left(t\right)\right)\right)\right.\\ \;\left.+\left[f\left({\bar{x}}_{c}\left(t\right)\right)-f\left(x_{c}\left(t\right)\right)\right]+\left[f\left(x_{c}\left(t\right)\right)-f\left({\bar{x}}_{c}\left(t\right)\right)\right]\right\} \end{array}$$

$$\begin{array}{c} \geq 1+\frac{\varepsilon_{1}}{k_{i}^{\left(1\right)}}\left\{\left[f\left({\bar{x}}_{c}\left(t\right)\right)-f\left(x_{c}\left(t\right)\right)\right]+\left[f\left(x_{c}\left(t\right)\right)-f\left({\bar{x}}_{c}\left(t\right)\right)\right]\right\}\\ \;+\frac{\varepsilon_{2}}{k_{i}^{\left(2\right)}}\left\{k_{i}^{\left(2\right)}f\left(x_{i}\left(t\right)\right)+\left[f\left({\bar{x}}_{c}\left(t\right)\right)-f\left(x_{c}\left(t\right)\right)\right]+\left[f\left(x_{c}\left(t\right)\right)-f\left({\bar{x}}_{c}\left(t\right)\right)\right]\right\}\\ \;+\frac{\varepsilon_{3}}{k_{i}^{\left(3\right)}}\left\{\left[f\left({\bar{x}}_{c}\left(t\right)\right)-f\left(x_{c}\left(t\right)\right)\right]+\left[f\left(x_{c}\left(t\right)\right)-f\left({\bar{x}}_{c}\left(t\right)\right)\right]\right\}. \end{array}$$

Since $f(x_{c}\left(t\right))<0$, we have:

$$\begin{array}{cc} \; & 1+\frac{\varepsilon_{1}}{k_{i}^{\left(1\right)}}\left\{\left[f\left({\bar{x}}_{c}\left(t\right)\right)-f\left(x_{c}\left(t\right)\right)\right]+\left[f\left(x_{c}\left(t\right)\right)-f\left({\bar{x}}_{c}\left(t\right)\right)\right]\right\}\\ \; & \;+\frac{\varepsilon_{2}}{k_{i}^{\left(2\right)}}\left\{k_{i}^{\left(2\right)}f\left(x_{i}\left(t\right)\right)+\left[f\left({\bar{x}}_{c}\left(t\right)\right)-f\left(x_{c}\left(t\right)\right)\right]+\left[f\left(x_{c}\left(t\right)\right)-f\left({\bar{x}}_{c}\left(t\right)\right)\right]\right\}\\ \; & \;+\frac{\varepsilon_{3}}{k_{i}^{\left(3\right)}}\left\{\left[f\left({\bar{x}}_{c}\left(t\right)\right)-f\left(x_{c}\left(t\right)\right)\right]+\left[f\left(x_{c}\left(t\right)\right)-f\left({\bar{x}}_{c}\left(t\right)\right)\right]\right\}\\ \; & \geq 1+\frac{\varepsilon_{1}}{k_{i}^{\left(1\right)}}f\left(x_{c}\left(t\right)\right)+\frac{\varepsilon_{2}}{k_{i}^{\left(2\right)}}f\left(x_{c}\left(t\right)\right)+\frac{\varepsilon_{3}}{k_{i}^{\left(3\right)}}f\left(x_{c}\left(t\right)\right)\\ \; & =1+\left(\frac{\varepsilon_{1}}{k_{i}^{\left(1\right)}}+\frac{\varepsilon_{2}}{k_{i}^{\left(2\right)}}+\frac{\varepsilon_{3}}{k_{i}^{\left(3\right)}}\right)f\left(x_{c}\left(t\right)\right)\\ \; & =1+\left(\frac{\varepsilon_{1}}{k_{i}^{\left(1\right)}}+\frac{\varepsilon_{2}}{k_{i}^{\left(2\right)}}+\frac{\varepsilon_{3}}{k_{i}^{\left(3\right)}}\right)4x_{c}\left(t\right)\left(1-x_{c}\left(t\right)\right)\\ \; & =1-\left(\frac{\varepsilon_{1}}{k_{i}^{\left(1\right)}}+\frac{\varepsilon_{2}}{k_{i}^{\left(2\right)}}+\frac{\varepsilon_{3}}{k_{i}^{\left(3\right)}}\right)4x_{c}\left(t\right)\left(x_{c}\left(t\right)-1\right). \end{array}$$

Furthermore, from Equation (12), we have:

$$1+\left(\frac{\varepsilon_{1}}{k_{i}^{\left(1\right)}}+\frac{\varepsilon_{2}}{k_{i}^{\left(2\right)}}+\frac{\varepsilon_{3}}{k_{i}^{\left(3\right)}}\right)f\left(x_{c}\left(t\right)\right)\leq -1.$$

Additionally, considering Equation (10) and $x_{c}\left(t\right)\geq R$, we obtain:

$$1-\left(\frac{\varepsilon_{1}}{k_{i}^{\left(1\right)}}+\frac{\varepsilon_{2}}{k_{i}^{\left(2\right)}}+\frac{\varepsilon_{3}}{k_{i}^{\left(3\right)}}\right)4x_{c}\left(t\right)\left(x_{c}\left(t\right)-1\right)\leq 1-\left(\frac{\varepsilon_{1}}{k_{i}^{\left(1\right)}}+\frac{\varepsilon_{2}}{k_{i}^{\left(2\right)}}+\frac{\varepsilon_{3}}{k_{i}^{\left(3\right)}}\right)4R\left(R-1\right).$$

To ensure that $x_{c}\left(t+1\right)\leq -1$, it follows that:

$$\begin{array}{cc} 1-\left(\frac{\varepsilon_{1}}{k_{i}^{\left(1\right)}}+\frac{\varepsilon_{2}}{k_{i}^{\left(2\right)}}+\frac{\varepsilon_{3}}{k_{i}^{\left(3\right)}}\right)4R\left(R-1\right) & \leq -1\\ ⟹\left(\frac{\varepsilon_{1}}{k_{i}^{\left(1\right)}}+\frac{\varepsilon_{2}}{k_{i}^{\left(2\right)}}+\frac{\varepsilon_{3}}{k_{i}^{\left(3\right)}}\right)4R\left(R-1\right) & \geq 2. \end{array}$$

Solving the above inequality yields:

$$R\geq R_{threshold}\approx \frac{1}{2}\left(1+\sqrt{\frac{2}{\frac{\varepsilon_{1}}{k_{i}^{\left(1\right)}}+\frac{\varepsilon_{2}}{k_{i}^{\left(2\right)}}+\frac{\varepsilon_{3}}{k_{i}^{\left(3\right)}}}}\right).$$

To characterize the overall cascade failure process of the network, the average generalized degree of each order in the network is used to represent the generalized degree of individual nodes. Consequently, an approximate value of the perturbation threshold can be obtained as follows:

$$R_{threshold}\approx \frac{1}{2}\left(1+\sqrt{\frac{2k_{avg}^{\left(1\right)}k_{avg}^{\left(2\right)}k_{avg}^{\left(3\right)}}{\varepsilon_{1}k_{avg}^{\left(2\right)}k_{avg}^{\left(3\right)}+\varepsilon_{2}k_{avg}^{\left(1\right)}k_{avg}^{\left(3\right)}+\varepsilon_{3}k_{avg}^{\left(1\right)}k_{avg}^{\left(2\right)}}}\right).$$

Here, $k_{avg}^{\left(m\right)}\left(m=1,2,3\right)$ denotes the average generalized degree of *m*-order simplicial structures.

**Figure 12:**
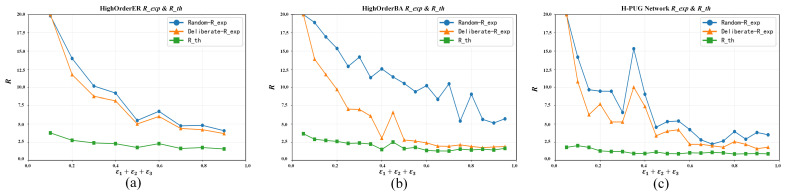
Shows the experimental averages and theoretical approximations of the perturbation threshold for third-order random networks (**a**), scale-free networks (**b**,**c**) H-PUG network. In the figure, the green line represents the theoretical approximation $R_{th}$, while the blue and orange lines correspond to the experimental averages under random attacks ($\mathrm{Random}\text{-}R_{exp}$) and targeted attacks ($\mathrm{Deliberate}\text{-}R_{exp}$), respectively.

Figure 12 presents a comparison between the experimental averages and theoretical approximations of the perturbation thresholds under different attack strategies for synthetic third-order random networks (Figure 12a), scale-free networks (Figure 12b), and the H-PUG network from Section 3.2 (Figure 12c). In the figure, the horizontal axis represents the sum of the coupling strengths, and the vertical axis represents the perturbation threshold. The comparison reveals that for relatively small $\sum_{i=1}^{3}\varepsilon_{i}$, the average error between the theoretical and experimental perturbation thresholds *R* is relatively large. However, as the sum of the coupling strengths increases, the theoretical approximations align well with the experimental averages. The sources of these discrepancies can be attributed to two factors. First, the experimental averages are taken over both random and targeted attacks, particularly in scale-free and H-PUG networks where node generalized degrees vary widely. Second, each realization of the synthetic network differs, and the evolutionary dynamics of third-order networks inherently include stochasticity.

## 5. Conclusions

In this study, based on the structural characteristics of simplicial networks and combining the coupled map lattice (CML) theory, we firstly propose a model suitable to describe cascading failures in *m*-order simplicial complex networks—the Higher-Order CML model. When m=1, this model reduces to the standard CML cascading failure model for ordinary networks, making it applicable both for ordinary networks and for simulating the cascading failure processes of *m*-order simplicial complex networks. Furthermore, we construct fourth-order simplicial networks based on random and scale-free networks to analyze the characteristics of cascading failures in fourth-order simplicial networks and to compare the robustness among higher-order structures of different dimensions. Building on this, higher-order structures of different dimensions are incorporated into ordinary networks, and the conclusions are validated through robustness experiments and empirical network analysis. Finally, an explicit expression for the perturbation threshold causing global failure in third-order simplicial complex networks is derived.

The results show that fourth-order simplicial networks are robust against random failures but vulnerable to targeted attacks, regardless of the underlying network type. Moreover, increasing the simplicial dimension enhances structural robustness, leading to higher critical perturbation thresholds and slower cascade propagation. When the same number of higher-order structures is introduced, networks with higher simplicial dimensions exhibit stronger resilience. The analytical perturbation threshold further reveals a negative correlation between the critical perturbation and the total coupling strength among structural components. These findings are consistently supported by numerical simulations and empirical network experiments.

Owing to differences in network construction, higher-order simplicial networks generated under the two topological structures have different network sizes, even when they contain the same number of simplicial structures, leading to distinct average generalized degrees at each order. As a result, direct comparisons of cascading failure evolution across different topologies under identical attack strategies are not meaningful, and such comparisons are therefore not pursued. In addition, the theoretical expression for the perturbation threshold is derived only for third-order simplicial networks. While this result mainly captures the coupling properties of third-order simplicial interactions and cannot fully describe more complex higher-order mechanisms, it provides a useful basis for understanding and extending perturbation thresholds in higher-order simplicial networks. Extensions to higher orders may exhibit quantitative deviations from numerical simulations, but the qualitative trends and critical behaviors are expected to remain consistent.

Overall, it should be emphasized that the conclusions drawn in this study are, to some extent, dependent on the chosen model assumptions and parameter settings. And this study demonstrates that simplicial dimension plays a fundamental role in shaping cascading dynamics and robustness in higher-order networks. The proposed higher-order CML framework provides a unified perspective for further enhancing the descriptive power of higher-order simplicial network models, and enriching the understanding of cascading failure mechanisms.

At the same time, several issues deserve further investigation. In particular, extending the perturbation-threshold analysis to more general higher-order simplicial interactions, incorporating weighted, directed, time-varying, and heterogeneous higher-order couplings, as well as considering internal interactions within higher-order structures, may further improve the realism and generality of the proposed framework. In addition, validating the model on larger real-world higher-order networks and exploring the interplay between higher-order topology, synchronization dynamics, and cascading propagation under different nonlinear dynamics remain important directions for future research. Such extensions would further enrich the understanding of cascading failure mechanisms and provide more realistic theoretical support for robustness analysis in real-world complex systems.

## Figures and Tables

**Table 1:** Simulation Parameters and Values for Cascading Failures of Higher-Order Structures.

Parameter	Definition	Value
$k_{0}$	Number of nodes in a simplex	$k_{0}=2,3,4,5$
f(x)	The Chaotic Logistic map	f(x)=4x(1−x)
$\varepsilon_{i}$	Coupling strength	$\varepsilon_{i}\in \left(0,1\right),\;i=1,2,3,4$
$p_{m}$	The generation probability of *m*-order simplicial structures in the network	$p_{m}\in \left(0,1\right),\;m=1,2,3,4$
*t*	Time steps for network evolution	20
*s*	Time of external perturbation	6
*R*	Perturbation threshold	[0,50]

**Table 2:** Simulation Parameters and Values for Cascading Failures in Random Higher-Order Networks.

Parameter	Definition	Value
*N*	Total number of nodes in the network	200
*M*	Number of simplices in the network	500

**Table 3:** Simulation Parameters and Values for Cascading Failures in Scale-Free Higher-Order Structures.

Parameter	Definition	Value
*N*	Total number of nodes in the network	500
*M*	Number of simplices in the network	$(N-k_{0}+1)\approx 500$

**Table 4:** Number of simplicial structures of different orders in the higher-order Power-US-Grid network.

Simplex	The Number of H-PUG/12H-PUG/13-HPUG Network
1-Simplex	5223/5223/6350
2-Simplex	414/588/0
3-Simplex	45/0/45

## Data Availability

The original contributions presented in this study are included in the article. Further inquiries can be directed to the corresponding author.

## References

[1] Shu Y.H., Guo J.L., Qin S.Y. (2023). Research on the Characteristics of Airline Network Structure. Softw. Guide.

[2] Hu F., Zhao H.X., He J.B., Li F.X., Li S.L., Zhang Z.K. (2013). An evolving model for hypergraph-structure-based scientific collaboration networks. Acta Phys. Sin..

[3] Wang X.F., Xu J. (2004). Cascading failures in coupled map lattices. Phys. Rev. E.

[4] Xu J., Wang X.F. (2005). Cascading failures in scale-free coupled map lattices. Proceedings of the 2005 IEEE International Symposium on Circuits and Systems (ISCAS).

[5] Zhou D.Y., Hu F.N., Wang S.L., Chen J. (2021). Power network robustness analysis based on electrical engineering and complex network theory. Phys. A Stat. Mech. Its Appl..

[6] Baggio J.A., BurnSilver S.B., Arenas A., Magdanz J.S., Kofinas G.P., De Domenico M. (2016). Multiplex social ecological network analysis reveals how social changes affect community robustness more than resource depletion. Proc. Natl. Acad. Sci. USA.

[7] Motter A.E., Lai Y.C. (2002). Cascade-based attacks on complex networks. Phys. Rev. E.

[8] Wang S.L., Lv W.Z., Zhang J.H., Luan S.Y., Chen C., Gu X.F. (2021). Method of power network critical nodes identification and robustness enhancement based on a cooperative framework. Reliab. Eng. Syst. Saf..

[9] Battiston F., Cencetti G., Iacopini I., Latora V., Lucas M., Patania A., Young J.G., Petri G. (2020). Networks beyond pairwise interactions: Structure and dynamics. Phys. Rep..

[10] Wang Z., Zeng G., Yin Q., Guo L., Hong Z. (2026). Coupled Dynamics of Vaccination Behavior and Epidemic Spreading on Multilayer Higher-Order Networks. Entropy.

[11] Benson A.R., Gleich D.F., Higham D.J. (2021). Higher-order network analysis takes off, fueled by classical ideas and new data. arXiv.

[12] Zhang Y.J., Gao Y.H., Wang Y.T., Yang C., Liu X.Y., Wang W. (2026). Awareness tames abrupt transitions in higher-order epidemics. Chaos Solitons Fractals.

[13] Song J.Y., Li W.J., Xiao Y.Z., Chen L., Yang C., Qi L., Wang W. (2026). Optimal information spreading strategy for containing epidemic spreading on higher-order multiplex networks. Appl. Math. Comput..

[14] Gao Y.H., Li J.C., Gao F., Wang W. (2026). Coevolution of multipathogens on higher-order networks. Chaos Solitons Fractals.

[15] Millán A.P., Torres J.J., Bianconi G. (2020). Explosive higher-order Kuramoto dynamics on simplicial complexes. Phys. Rev. Lett..

[16] Aguiar M., Bick C., Dias A. (2023). Network dynamics with higher-order interactions: Coupled cell hypernetworks for identical cells and synchrony. Nonlinearity.

[17] Iacopini I., Petri G., Barrat A., Latora V. (2019). Simplicial models of social contagion. Nat. Commun..

[18] Matamalas J.T., Gómez S., Arenas A. (2020). Abrupt phase transition of epidemic spreading in simplicial complexes. Phys. Rev. Res..

[19] Billings J.C.W., Hu M., Lerda G., Medvedev A.N., Mottes F., Onicas A., Santoro A., Petri G. (2019). Simplex2vec embeddings for community detection in simplicial complexes. arXiv.

[20] Ma X.J., Zhao H.X., Hu F. (2016). Cascading failure analysis in hyper-network based on the hypergraph. Acta Phys. Sin..

[21] Peng H., Zhao Y.F., Zhao D.D., Zhong M., Hu Z.L., Han J.M., Li R.C., Wang W. (2023). Robustness of higher-order interdependent networks. Chaos Solitons Fractals.

[22] He J.Y., Zeng A. (2023). Link cascade failure in directed networks with higher-order structures. Phys. Lett. A.

[23] Yu W.Q., Ma F.X., Chen Y., Ma X.J. (2025). Robustness Analysis of Higher-Order Networks Based on Adaptation. Complex Syst. Complex. Sci..

[24] Bo L., Zeng Y.J., Yang R.M., Lu L.Y. (2024). Fundamental statistics of higher-order networks: A survey. Acta Phys. Sin..

[25] Bick C., Gross E., Harrington H.A., Schaub M.T. (2023). What are higher-order networks?. SIAM Rev..

[26] Gambuzza L.V., Di Patti F., Gallo L., Lepri S., Romance M., Criado R., Frasca M., Latora V., Boccaletti S. (2021). Stability of synchronization in simplicial complexes. Nat. Commun..

[27] Lucas M., Cencetti G., Battiston F. (2020). Multiorder Laplacian for synchronization in higher-order networks. Phys. Rev. Res..

